# Analysis of Differential Diagnosis of Benign and Malignant Partially Cystic Thyroid Nodules Based on Ultrasound Characterization With a TIRADS Grade-4a or Higher Nodules

**DOI:** 10.3389/fendo.2022.861070

**Published:** 2022-05-16

**Authors:** Chen-Yi Wang, Yang Li, Meng-Meng Zhang, Zhi-Long Yu, Zi-Zhen Wu, Chen Li, Dong-Chen Zhang, Ying-Jiang Ye, Shan Wang, Ke-Wei Jiang

**Affiliations:** ^1^ Department of Gastroenterological Surgery, Peking University People’s Hospital, Beijing, China; ^2^ Laboratory of Surgical Oncology, Beijing Key Laboratory of Colorectal Cancer Diagnosis and Treatment Research, Peking University People’s Hospital, Beijing, China; ^3^ Thyroid Surgery Department, The First Affiliated Hospital of Zhengzhou University, Zhengzhou, China

**Keywords:** thyroid neoplasms, thyroid nodule, ultrasonography, factor analysis, statistical, partially cystic thyroid nodules

## Abstract

Partially cystic thyroid nodules (PCTNs) are a kind of thyroid nodule with both solid and cystic components, and are usually misdiagnosed as benign nodules. The objective of this study was to determine the ultrasound (US) characterizations with a TIRADS Grade-4a or higher partially cystic thyroid nodules (PCTNs) which are associated with being malignant or benign. In this study, 133 PCTNs with a TIRADS Grade-4a or higher were enrolled; 83 were malignant and 50 were benign. TI-RADS classification can detect malignant PCTNs, and its sensitivity, specificity, positive predictive value, negative predictive value, and accuracy are 39.8%, 96.0%, 94.3%, 49.0%, and 60.9%, respectively. Univariate analyses revealed that nodule shape, margin, and structure were related to PCTNs’ being benign and malignant, among which nodules taller-than-wide, with an irregular shape, non-smooth margin, eccentric sharp angle, or edge sharp angle were significantly associated with malignancy while ovoid to round nodules, smooth margins, multiple separation, and eccentric obtuse angle structures were significantly associated with a benign nature. For the solid part of PCTNs, its free margin, echo, and calcification are related to benign and malignant PCTNs. Among them, the free margin of the solid part is non-smooth, hypoechoic, and microcalcification, which are related to malignant PCTNs, while the free margin of the solid part is smooth, isoechoic, macrocalcification, non-calcification and are related to benign PCTNs. Calcification of solid part and free margin are important factors for predicting malignant PCTNs. In addition, nodules’ composition, blood flow signal, and other factors had nothing to do with PCTNs’ being benign or malignant. In the multivariate Logistic regression analysis, solid part calcification (OR: 17.28; 95%CI: 5.14~58.08) and free margin (OR: 3.18; 95%CI: 1.01~10.00) were revealed to be the strongest independent predictors for malignancy (P<0.05). Our study indicated that understanding the ultrasound characteristics of malignant PCTNs, to avoid misdiagnosed PCTNs patients, is important to make a precise diagnosis and prognosis of PCTNs.

## Introduction

Partially cystic thyroid nodules (PCTNs) are a kind of thyroid nodule with both solid and cystic components, accounting for approximately 20.9% of thyroid nodules (1). Since most PCTNs are potentially benign lesions induced by degeneration, such as nodular hyperplasia, the management of partially cystic thyroid nodules is often underrated. However, PCTNs still have a 3.3% to 17.6% rate of malignancy, and the incidence of malignancy is not related to the proportion of cystic components (2). Therefore, it is of great importance to identify sonographic features that distinguish malignant PCTNs in clinical practice.

Fine-needle aspiration biopsy (FNAB) is a reliable and cost-efficient method to evaluate thyroid nodules, with an overall accuracy of 98% and a false-negative rate below 5%. Current guidelines in China only consider the size (1.0-1.5 cm) as a criterion for FNAB, regardless of the cystic portion (3). Similar guidelines from the American Thyroid Association consider a size ≥1 cm (in nodules with high suspicion), 1.5–2.0 cm (in nodules with any suspicious US features), or 2.0 cm (in nodules without any suspicious US features) as the threshold size (4). However, its reliability in cystic thyroid is not clear. In cystic thyroid malignancy, performing a diagnostic FNAB is challenging due to the lack of cellularity in cystic areas, presence of nuclear debris, and histiocyte/macrophages (5), which frequently lead to misdiagnosis of a benign cystic nodule.

Thyroid ultrasonography(US) is an essential diagnostic technique for assessing the malignancy risk of thyroid nodules (6, 7), but there are few studies on the ultrasound characteristics of PCTNs diagnosis and prediction, and previous studies showed that the number of malignant samples was too little, and the benign control group lacked postoperative pathological results. Shi et al. (8) reported that a taller-than-wide shape, eccentric configuration, non-smooth margin, hypoechogenicity, microcalcification, and spiculated or microlobulated margin were significantly associated with PCTNs malignancy. According to Kim et al. (9), eccentric configuration with an acute angle and microcalcifications can significantly increase the risk of malignancy. With PCTNs, it is difficult to distinguish between benign and malignant in clinical practice, and it is easy to misdiagnose as a benign condition. Hence, the aim of the present study was to investigate the benign and malignant ultrasound characteristics of PCTNs in order to help clinicians make accurate treatment choices. The results are reported below.

## 1 Materials and Methods

### 1.1 Patients

The study was approved by the Ethical Committee of the First Affiliated Hospital of Zhengzhou University in China and was performed in accordance with the Declaration of Helsinki for human studies. The requirement of informed consent from human subjects is sometimes waived by institutional review boards (IRBs) for protocols that include a retrospective review of images acquired for clinical diagnostic purposes. From January 2016 to January 2020, 133 PCTNs patients who underwent thyroidectomy surgery were retrospectively enrolled in this case-control study. The inclusion criteria were: 1) patients with mixed echoic thyroid nodules that were confirmed by our central ultrasound examination before surgery; 2) no invasive procedure such as thyroid surgery or FNA was previously performed; and 3) all PCTNs were suspicious for malignancy (preoperative TIRADS grade 4a and above) and underwent surgical resection. In addition, the exclusion criteria were as follows: 1) without PCTNs pathologic examinations after surgery and 2) the color Doppler report was not checked by the chief physician. Finally, 133 PCTNs patients were included in this study; there were 59 males and 74 females, ranging in age from 9 to 71 years old, with an average age of 46.32 ± 12.27 years. The longest diameter of the nodules is 9 to 48mm. According to Chinese Thyroid Imaging Reporting and Data System( C-TIRADS) (3), 98 patients were classified into score 4a (1 point, 2-10% malignancy rate), seven patients were classified into score 4b (2 points, 10-50% malignancy rate), 0 patients were classified into score 4c (3-4 points, 50-90% malignancy rate), and 28 patients were classified into score 5 (5 points, >90% malignancy rate). We defined score 4a as suspected malignant, and score 4b or higher was defined as malignant.

83 patients with postoperative pathological findings confirmed to be malignant were included in the malignant PCTNs group, and were made up of 80 papillary thyroid carcinomas and three follicular thyroid carcinomas. The remaining 50 patients with benign PCTNs were included in the case-control group, and included 44 cases of nodular goiter, four cases of Hashimoto’s thyroiditis, and two cases of thyroid adenoma.

### 1.2 Methods

#### 1.2.1 Equipment

Images were acquired using a Toshiba Aplio^®^ XG ultrasound scanner (Toshiba, Japan) connected to an 8-14 MHz linear transducer (PLT 1202S probe). Patients took the supine position to fully expose the neck. Ultrasonography was performed by two attending physicians in the same medical group with more than 8 years of experience in thyroid ultrasonography (>2000 cases/year), and the results were checked by one chief physician who had 24 years of experience in thyroid ultrasonography (>4000 cases/year) to prevent any artificial bias and ensure optimized image quality. The depth, gain, and focus of scanning were adjusted according to physical status after locating the thyroid nodules, and then clear 2D images were displayed and stored. Then, color Doppler ultrasound was applied to inspect the thyroid nodules and surrounding tissues, so as to determine the blood flow inside the foci and the surrounding tissues. The range was set to 9.8 cm/s, the frame rate was 8 frames/s, the color gain was 50 dB, and the filter was 6.

#### 1.2.2 Image Analysis

**Figure 1:**
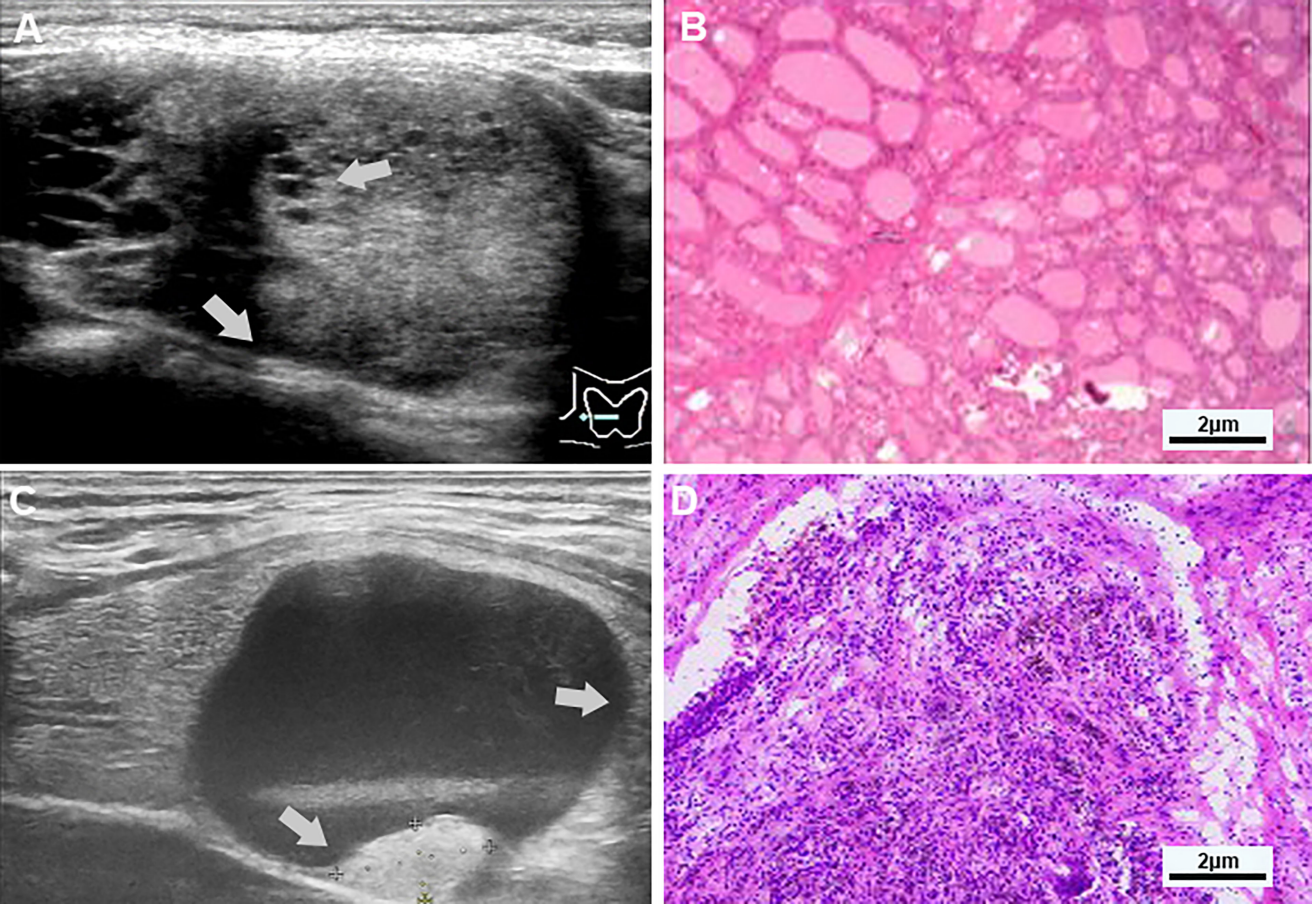
**(A, B)** Examples of typical ultrasonographic and pathological sections of multiple separation partially cystic thyroid nodules. Transverse section of a cystic-solid nodule of the thyroid, mainly solid, with smooth and irregular margins, and an acute angle between the multiple-separated and fused solid components and the nodule wall (indicated by gray-white arrows), for the hypoechoic, the free edge is non-smooth, and the pathology confirmed nodular goiter. **(C, D)** Examples of typical ultrasonographic and pathological sections of predominant cystic partially cystic thyroid nodules. Longitudinal section of a cystic-solid nodule of the thyroid, mainly cystic, with smooth, oval nodule margins, and an obtuse angle between the solid part attached to the posterior wall and the nodule wall (indicated by the gray-white arrow), For isoechoic, the free edge is smooth, and the pathology confirmed nodular goiter with cystic degeneration, close to the thyroid membrane.

**Figure 2:**
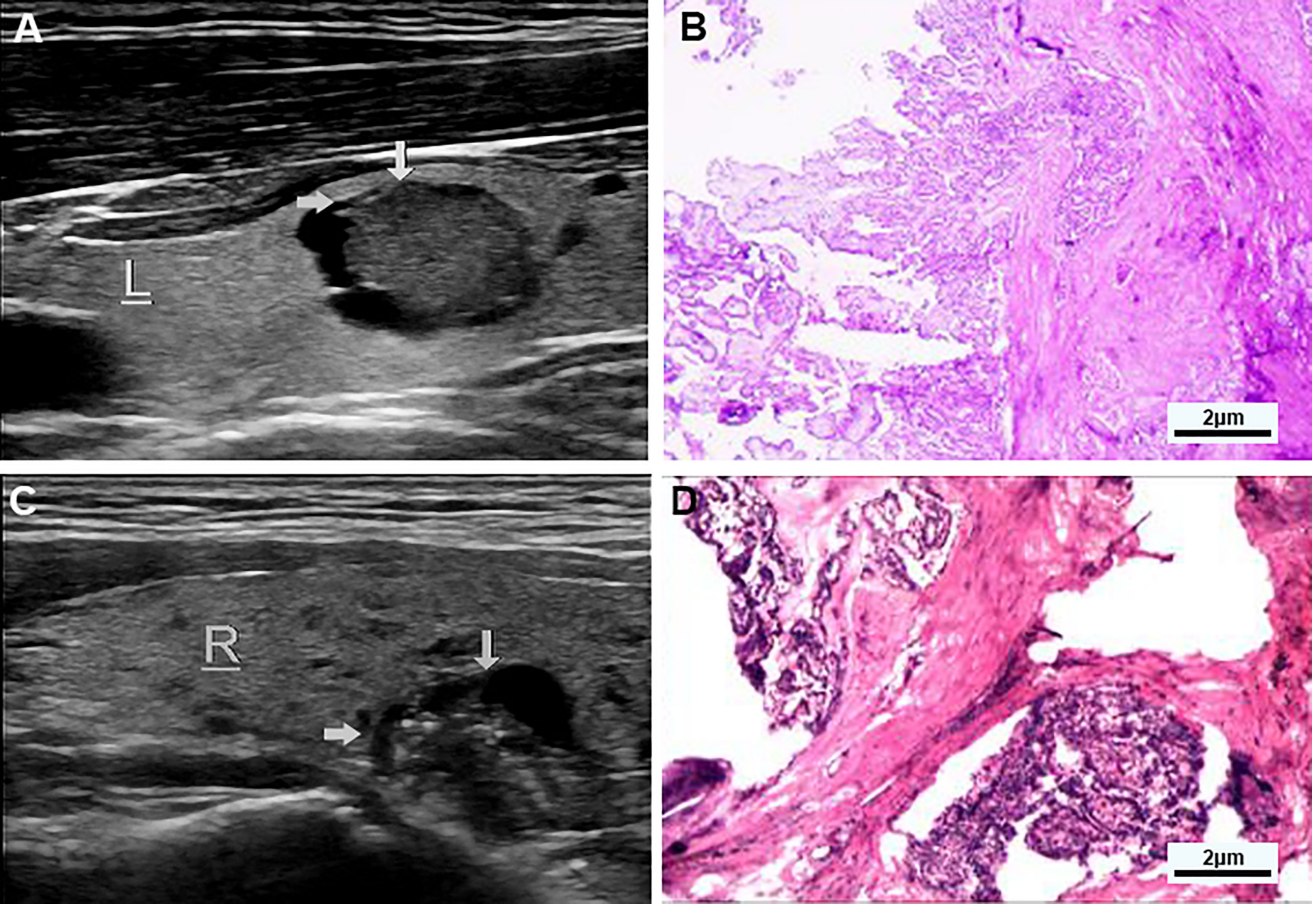
**(A, B)** Examples of typical ultrasonographic and pathological sections of predominant solid partially cystic thyroid nodules. Longitudinal section of a cystic-solid nodule of the thyroid, mainly solid, with smooth and irregular margins, and an acute angle between the solid part of the anterior wall and the nodule wall (indicated by the gray arrow). It is hypoechoic, and the free edge is not smooth. TI-RADS grade 5 and the pathology confirmed papillary thyroid carcinoma, invading surrounding tissues. **(C, D)** Examples of typical ultrasonographic and pathological sections of macrocalcification cystic thyroid nodules. Longitudinal section of a cystic-solid nodule of the thyroid gland, mainly cystic, with smooth and irregular borders, and an acute angle between the solid part attached to the posterior wall and the nodule wall (indicated by the gray arrow) has multiple strong echogenic light spots, which are isoechoic, and the free edge is not smooth, and the pathology confirmed papillary thyroid carcinoma.

US images in our study were retrospectively evaluated by two experienced physicians (each with more than 8 years of experience in thyroid ultrasonography) who were blinded to the other imaging results and clinical as well as histopathological data. Two physicians performed the following US finding for each entire nodule: size (the longest diameter), internal content (predominantly solid vs. predominant cystic), shape (ovoid to round vs. irregular vs. taller-than-wide), margin (smooth vs. non-smooth), and structure (eccentric acute angle, edge acute angle, eccentric obtuse angle, multiple separation). The internal solid portion inspection includes: free margin(smooth vs. non-smooth), echo (hypoechoic, isoechoic, and hyperechoic), calcification (microcalcification, macrocalcification, none), and blood flow signal level (0, 1, 2, 3). The ultrasound results report uses the 2020 Chinese thyroid nodule ultrasound malignant tumor risk stratification guideline C-TIRADS recommended term (3): hyperechoic refers to the echogenicity is higher than that of the surrounding thyroid parenchyma, hypoechoic means the echogenicity is similar to that of the surrounding thyroid parenchyma, isoechoic refers to the echogenicity that is similar to that of the surrounding thyroid parenchyma. Predominately solid means solid components accounted for more than 50% of the nodules (**Figure 1**). Predominately cystic refers to solid components that accounted for <50% of the nodules (**Figure 2**). Spongiform refers to multiple tiny cystic spaces that occupy the entire nodules without aggregated solid tissues; multiple separation is defined as the inner solid portion bordered by the interface between the cystic and solid components (**Figure 1**); microcalcifications are defined as punctate echogenic foci of less than about 1 mm with or without shadowing; macrocalcififications are defined as the echogenic foci that are larger than 1 mm, usually accompanied by posterior shadowing (**Figure 2**). The class of blood flow signal was graded according to the Adler semi-quantitative method: grade 0, no blood flow; grade 1, little blood flow; grade 2, moderate blood flow; grade 3, abundant blood flow.

### 1.3 Statistical Methods

All analyses were conducted using SPSS version 24.0 (IBM SPSS Statistics for Macintosh, Version 24.0). Normally distributed enumeration data were expressed as cases and percentages (%), measurement data were expressed as mean ± standard deviation ( $\bar{x}\pm s$ ), continuous variables were compared by t-test, and categorical variables were compared by χ2 test. The multivariate analysis was performed by binary logistic regression analysis. Because nodule sizes were distributed non-normally, the Mann-Whitney U test was chosen. Statistical significance was established at P<0.05.

## 2 Results

According to the C-TIRADS (3) in the 2020 Chinese Thyroid Nodules Ultrasound Malignancy Risk Stratification Guidelines, there were 98 grade 4a nodules, seven grade 4b nodules, and 28 grade 5 nodules in this study. 98 cases were considered suspected malignant(TI-RADS 4a), and 35 cases were considered malignant (TI-RADS 4b or higher). According to preoperative ultrasound and postoperative pathological results, 33 true positive, 48 true negative, two false positive, and 50 false-negative(n=133) were detected, and the sensitivity, specificity, positive predictive value, negative predictive value, and accuracy were respectively 39.8% (33/83), 96.0% (48/50), 94.3% (33/35), 49.0% (48/98), and 60.9% (81/133).

Univariate analyses of US features of 133 PCTNs with TIRADS grade-4a or higher were summarized in **Table 1**. According to the postoperative pathological results, the prevalence of malignant PCTNs was 83 of 133(62.4%) and the prevalence of benign PCTNs was 50 of 133(37.6%). There was no significant gender difference between benign and malignant PCTNs patients. The average age of malignant PCTNs patients was smaller than that of benign PCTNs patients [(44.14 ± 11.59) years vs. (49.92 ± 12.62) years]. The TI-RADS grade of malignant PCTNs patients was higher than that of benign PCTNs patients. The difference was statistically significant (P<0.01, t=20.70).

**Table 1:** Basic characteristics of the patients.

Essential information	Malignancy (n = 83)	Benign condition (n =50)	t/χ^2^	P
Gender (%)			0.43	0.51
Male	35 (42.2%)	24 (48.0%)		
Female	48 (57.8%)	26 (52.0%)		
Age (year, Mean ± SD)	44.14 ± 11.59	49.92 ± 12.62	-2.64	0.01
TI-RADS grade (%)			20.70	<0.01
4a	50 (60.2%)	48 (96.0%)		
4b	7 (8.4%)	0 (0.0%)		
5	26 (31.3%)	2 (4.0%)		

Ultrasound features of PCTNs found that nodule shape, margin, and structure were associated with benign and malignant PCTNs, among which taller-than-wide, irregular shape, non-smooth margin, and structure with eccentric/edge acute angle were significantly associated with malignant PCTNs, but ovoid to round shape, smooth margin, eccentric obtuse angle, and multiple separation structures were associated with benign PCTNs. For the solid part of PCTNs, the free edge, echo, and calcification of PCTNs are related to benign and malignant PCTNs. The free edge of the solid part being non-smooth, hypoechoic, and microcalcification are related to malignant PCTNs, while the free edge of solid part being smooth and isoechoic, macrocalcification, and non-calcification are related to benign PCTNs. The above results were statistically significant (P < 0.01) (**Table 2**). In addition, we found that nodule composition (P=0.83) and blood flow signal (P=0.20) were not associated with benign or malignant PCTNs.

**Table 2:** Ultrasound characteristics of the partial cystic nodules of the thyroid gland.

Ultrasonic feature	Pathological results		χ^2^	P
	Malignancy (n = 83)	Benign (n = 50)		
Composition (%)			0.49	0.83
Predominant cystic	4 (4.8%)	2 (4.0%)		
Predominant solid	79 (95.2%)	48 (96.0%)		
Shape (%)			15.76	<0.01
Ovoid to round	24 (28.9%)	32 (64.0%)		
Irregular	55 (66.3%)	18 (36.0%)		
Taller-than-wide	4 (4.8%)	0 (0.0%)		
Margin (%)			18.38	<0.01
Smooth	33 (39.8%)	39 (78.0%)		
Non-smooth	50 (60.2%)	11 (22.0%)		
Structure (%)			7.50	<0.01
Eccentric sharp angle	69 (83.1%)	36 (72.0%)		
Edge sharp Angle	8 (9.6%)	2 (4.0%)		
Multiple separation	6 (7.2%)	8 (16.0%)		
Eccentric obtuse angle	0 (0.0%)	4 (8.0%)		
Free margin (%)			12.96	<0.01
Smooth	18 (21.7%)	26 (52.0%)		
Non-smooth	65 (78.3%)	24 (48.0%)		
Echo (%)			10.43	<0.01
Isoechoic	0 (0.0%)	6 (12.0%)		
Hypoechoic	83 (100%)	44 (88.0%)		
Calcification (%)			43.85	<0.01
Non-calcification	6 (7.2%)	24 (48.0%)		
Macrocalcification	75 (90.4%)	18 (36.0%)		
Macrocalcification	2 (2.4%)	8 (16.0%)		
Blood flow signal (%)			0.95	0.20
0	3 (3.6%)	0 (0.0%)		
1	27 (32.5%)	12 (24.0%)		
2	34 (41.0%)	28 (56.0%)		
3	19 (22.9%)	10 (20.0%)		

Multivariate Logistic regression analysis showed (**Table 3**) solid partial calcification (OR: 17.28; 95%CI: 5.14-58.08) and free margin (OR: 3.18; 95%CI: 1.01-10.00) were the predictors of malignant PCTNs (all P<0.05). Among them, microcalcification and non-smooth free margin were important factors for predicting malignant PCTNs, and the risk of microcalcification in ultrasound signs was the most significant, which was 17.28 (P<0.05) times higher than that of macrocalcification and non-calcification.

**Table 3:** Results of Logistic regression analysis of the ultrasound characteristics of partial cystic thyroid nodules.

Factor	β	SE	Wald	OR	95%CI	P
Free edge (smooth vs non-smooth)	1.16	0.59	3.92	3.18	1.01~10.00	0.05
Calcification (non-calcification & macrocalcification vs microcalcification)	2.85	0.62	21.22	17.28	5.14~58.08	<0.01

## 3 Discussion

TI-RADS is thyroid imaging reporting and data system for ultrasonography, mainly to classify the benign and malignant degree of thyroid nodules. Among them, the malignancy of TI-RADS 4a may be about 2-10%, TI-RADS 4b is up to 10-50%, TI-RADS 4c is 50-90% and TI-RADS 5 is >90% (3). PCTNs are easily misdiagnosed, especially for inexperienced radiologists, as their typical US characteristics differ from solid nodules, so the clinical management of PCTNs remains a challenge. In our study, the sensitivity of TI-RADS for PCTNs was 39.8% and the specificity was 96.0%, which was slightly different from the sensitivity of 72.7% and the specificity of 98.0% in similar studies (9). PCTNs whose ultrasound features are between benign and malignant are more difficult to differentiate. Compared with other related studies of solid thyroid nodules (10), the sensitivity and specificity of TI-RADS for differentiating benign and malignant thyroid nodules were 91.67% and 52.8%. Therefore, compared with solid nodules, PCTNs have a lower sensitivity and higher specificity for ultrasound diagnosis.

According to the analysis of the results of ultrasound images in this study, the solid component in some cystic nodules with eccentric sharp angle, edge sharp angle, non-smooth free edge, nodule hypoechoic, or microcalcification was associated with malignant PCTNs (P<0.01). A related report found that, compared with benign nodular goiters, PCTNs more frequently presented with calcification, hypoechogenicity of the solid part, hypoenhancement, heterogeneous enhancement, centrifugal perfusion, peak intensity index <1, time to peak index ≥1, and area under the curve index <1 on preoperative US and CEUS (11). A recent review included eight studies with a total of 2,004 PCTNs. Seven features were considered to be associated with malignancy. High specificity (>0.9) was found in nodules with a taller-than-wide shape, those that were spiculated/microlobulated or with an ill-defined margin, those with microcalcification, and a non-smooth rim. Among US features, eccentric configuration, microcalcification, and marked or mild hypoechogenicity were more reliable in predicting malignancy (AUC: 0.9592, 0.8504, and 0.8092, respectively) (2). We also found that the nodules that were ovoid to round, with a smooth free margin in solid parts, isoechoic, macrocalcification, and non-calcification were also associated with benign PCTNs (P<0.01).

A domestic study in 2014 showed that the free margin of the solid part of PCTNs was divided into two groups: smooth and non-smooth. In this study, our findings are consistent with the above conclusions. We can explain this phenomenon by the fact that the tumor growth is heterogeneous, invasive, migratory, and has no complete capsule formation.

In most TI-RADS grading systems, the proportion of solid parts in the nodule and the blood flow signal are used as independent predictors in the risk assessment of thyroid nodules (12). However, the present study found no statistical difference between PCTNs with predominantly cystic and predominantly solid PCTNs in benign and malignant nodules, nor with the blood flow signal level. Therefore, we should not ignore nodules with predominantly cystic components and low blood flow signal grades.

Multivariate Logistic regression analysis showed that PCTNs solid part calcification and free margin were important factors for predicting malignant PCTNs, of which the non-smooth free margin and microcalcification in the solid part were poor factors for predicting malignant PCTNs, showing the key to predicting benign and malignant PCTNs. It is the observation of the general shape of the nodule(free margin) and the calcification of the solid part.

A suspicious FNAB is the most common indication for thyroidectomy. However, its reliability in large nodules is not clear. Osman described a lower sensitivity (55.5-71.4%) and higher false-negative rate (6.7-9.7%) in nodules larger than 4 cm, and only 17.3% patients were detected as having cancer (13, 14); meanwhile, PCTNs lack of cellularity in cystic areas, presence of nuclear debris, and histiocyte/macrophages (5) frequently lead to misdiagnosis of a cystic benign nodule. In the thyroid nodules we included in our study, all had cystic components, and the average diameter of the nodules was 3.2cm, therefore, compared with solid small nodules, the reference value of FNAB before surgery was relatively low. Published research (15) reported 119 patients with PCTNs who underwent thyroidectomy after FNA; 21 were malignant, yielding a 17.6% malignancy rate, and their team therefore recommended FNA for all PCTNs, however, the malignancy rate among PCTNs may have been overestimated due to selection bias for the enrolled patients. So, we consider the limitations of FNAB should be taken into consideration when making treatment decisions in PTCNs or nodules larger than 4 cm in size.

Not all surgeons are equally experienced in performing thyroid surgery. Studies have shown that surgeons who perform a large number of thyroidectomies each year have fewer complications than those who perform fewer operations. Such data do not mean that low-volume surgeons should not perform these operations. However, before undertaking a difficult operation, the surgeon should consider whether that patient might be better served by referral to a specialized institution for care. Meanwhile, in solitary small low-risk PTCNs, hemithyroidectomy, thanks to its lower complication rates, is still the safest standard of care, avoiding unnecessary morbidity (16).

This study has some limitations. First, the retrospective assessment of static ultrasound images of PCTNs in this study was less accurate than prospective dynamic ultrasound images. Secondly, the number of benign cases in this study is small, as patients are usually recommended surgical treatment when the TI-RADS grade is 4 or above in the ultrasound diagnosis. For nodules below grade 4, ultrasound follow-up or ultrasound-guided fine-needle aspiration is often recommended. Since this study did not use all PCTNs as candidates and only selected patients with surgical and pathological results, selection bias is inevitable for patients with only ultrasound results or puncture results. In addition, no hyperechoic nodules were found in this study, which may be a characteristic feature of benign PCTNs nodules.

## 4 Conclusion

TI-RADS can detect malignant PCTNs, and its sensitivity, specificity, positive predictive value, negative predictive value, and accuracy rate are 39.8%, 96.0%, 94.3%, 49.0%, and 60.9%, respectively. For PCTNs, nodule shape, margin, and structure were associated with benign and malignant PCTNs (P < 0.01). Among them, nodules taller-than-wide, with an irregular shape, non-smooth margin, eccentric sharp angle, and edge sharp angle were associated with malignant PCTNs, while ovoid to round nodules, smooth margins, multiple separation, and eccentric obtuse angle structures were associated with benign PCTNs. For the solid part of PCTNs, its free margin, echo, and calcification were related to benign and malignant PCTNs. Among them, the free margin of the solid part being non-smooth, hypoechoic, and microcalcification are related to malignant PCTNs, while the free margin of the solid part being smooth, isoechoic, macrocalcification, and non-calcification are related to benign PCTNs. The calcification of solid part and free margin are important factors for predicting malignant PCTNs. In addition, nodules with predominant cystic components and low blood flow signal levels cannot be ignored in clinical diagnosis and treatment. These findings are helpful for clinicians to more accurately identify benign and malignant PCTNs, avoid missing diagnosis and misdiagnosis of PCTNs patients, and are crucial for the clinical treatment and prognosis of PCTNs.

## Data Availability Statement

The raw data supporting the conclusions of this article will be made available by the authors, without undue reservation.

## Ethics Statement

The studies involving human participants were reviewed and approved by the Ethics Review Committee of First Affiliated Hospital of Zhengzhou University. Written informed consent from the participants’ legal guardian/next of kin was not required to participate in this study in accordance with the national legislation and the institutional requirements.

## Author Contributions

CW, YY, SW and KJ designed the study. CW, DZ and CL collected the clinical data, ultrasonography images and histopathological/cytopathology results. YL and MZ performed the statistical analysis. CW, ZY and ZW drafted and revised the manuscript. All authors contributed to the article and approved the submitted version.

## Funding

This study was supported by the National Nature Science Foundation of China (No. 81871962), National Scientific Center Project (No. 62088101) and the Industry-University-Research Innovation Fund in Ministry of Education of the People’s Republic of China (No.2018A01013).

## Conflict of Interest

The authors declare that the research was conducted in the absence of any commercial or financial relationships that could be construed as a potential conflict of interest.

## Publisher’s Note

All claims expressed in this article are solely those of the authors and do not necessarily represent those of their affiliated organizations, or those of the publisher, the editors and the reviewers. Any product that may be evaluated in this article, or claim that may be made by its manufacturer, is not guaranteed or endorsed by the publisher.
